# Role of Nuclear Factor Erythroid 2-Related Factor 2 in Diabetic Nephropathy

**DOI:** 10.1155/2017/3797802

**Published:** 2017-04-23

**Authors:** Wenpeng Cui, Xu Min, Xiaohong Xu, Bing Du, Ping Luo

**Affiliations:** ^1^Department of Nephrology, The Second Hospital of Jilin University, Changchun, Jilin 130041, China; ^2^Department of Gynaecology and Obstetrics, The Second Hospital of Jilin University, Changchun, Jilin 130041, China; ^3^Department of Cardiology, The First Hospital of Jilin University, Changchun, Jilin 130031, China

## Abstract

Diabetic nephropathy (DN) is manifested as increased urinary protein level, decreased glomerular filtration rate, and final renal dysfunction. DN is the leading cause of end-stage renal disease worldwide and causes a huge societal healthcare burden. Since satisfied treatments are still limited, exploring new strategies for the treatment of this disease is urgently needed. Oxidative stress takes part in the initiation and development of DN. In addition, nuclear factor erythroid 2-related factor 2 (Nrf2) plays a key role in the cellular response to oxidative stress. Thus, activation of Nrf2 seems to be a new choice for the treatment of DN. In current review, we discussed and summarized the therapeutic effects of Nrf2 activation on DN from both basic and clinical studies.

## 1. Introduction

According to the data from the International Diabetes Federation, the number of individuals who suffered from diabetic mellitus was 285 million in 2010, and this number will increase to 439 million in 2030. Diabetic nephropathy (DN), a serious complication of diabetic mellitus, is manifested as increased urinary protein level, decreased glomerular filtration rate (GFR), and final renal dysfunction [1]. DN is the leading cause of end-stage renal disease worldwide. Additionally, DN reduces patient's quality of life and increases the societal healthcare burdens. Various therapeutic approaches, such as glucose, lipid, and blood pressure control; renin-angiotensin system inhibitor application; and lifestyle changes, have been tried to slow the progression of DN [2]. However, the curative effect is not satisfactory, and the developing speed of DN is still the fastest among chronic kidney diseases (CKDs). Therefore, novel strategies for fighting against this disease are urgently needed.

The pathogenesis of DN is complicated. It is suggested that hemodynamic changes, genetic susceptibility, hyperglycemia, dyslipidemia, and oxidative stress take part in the progress and development of DN [3]. Among these issues, oxidative stress and its related inflammation and fibrosis have been reported to aggravate DN in recent years [4]. In our body, mechanisms maintaining the balance between oxidation and reduction are complicated. Nuclear factor erythroid 2-related factor 2 (Nrf2)/antioxidant-responsive element (ARE) pathway is proven to be crucial in this process [5, 6]. Therefore, activation of Nrf2/ARE pathway seems to be a potentially effective method for the treatment of DN. Here, we seek to review the contribution of Nrf2/ARE pathway activation to DN.

## 2. Oxidative Stress and DN

The nature of oxidative stress is the disproportionate generation of reactive oxygen species (ROS) and endogenous antioxidants. Low ROS level in body is crucial for survival and proliferation. High level of ROS leads to cellular apoptosis. Extremely high level of ROS gives rise to the damage of cellular macromolecules, including DNA [7], lipid [8], and protein [9]. It will cause mutations and followed by tissue injury or even necrotic cell death if those damages were not repaired [10].

Under diabetes condition, oxidative stress is triggered through a variety of ways, such as advanced glycation end-product accumulation and activation of polyol pathway, protein kinase C pathway, and renin angiotensin-aldosterone system. It is well accepted that oxidative stress takes part in the pathogenesis of DN. A large number of excellent reviews have shown that oxidative stress accelerates the progress of experimental DN [4, 11, 12]. For example, in type 1 diabetes rats, kidney expression of superoxide dismutase together with glutathione was obviously decreased, while restoring these two enzymes inhibited the progression of DN [13]. For type 2 diabetes rats, similarly, upregulation of malondialdehyde and downregulation of antioxidant enzymes, such as superoxide dismutase and glutathione, were observed in diabetes rat kidney. And, restoration of these enzymes halted hyperglycemia-induced oxidative stress and maintained renal function [14]. Moreover, it is not difficult to obtain similar evidence from clinical studies [15–17]. For example, in new published clinical trials, individuals who suffered from DN were enrolled. Kidney injury was attenuated accompanied by improving oxidative stress biomarkers (nitric oxide, glutathione, and malondialdehyde) after short-term supplementation of selenium or vitamin E [15, 16]. Taken together, antioxidative stress therapy might bring favorable outcomes to patients with DN.

## 3. Oxidative Stress and Nrf2/ARE Pathway

### 3.1. Components of Nrf2/ARE Pathway

**Figure 1:**
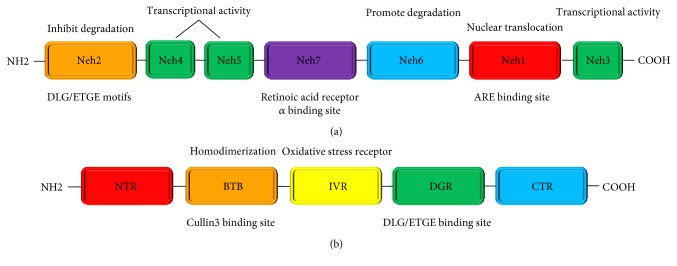
Molecular structure of Nrf2 (a) and Keap1 (b) proteins. (a) There are seven domains in the Nrf2 molecule, and a brief explanatory note for the main function of each domain was given. (b) There are five domains in the Keap1 molecule, and a brief explanatory note for the main function of each domain was given.

Nrf2 is a smart transcription factor with a basic leucine zipper motif [18]. It has seven highly conserved Nrf2-ECH homology domains, Neh1 to Neh7 [19, 20]. Neh1 domain includes a basic leucine zipper structure, which facilitates dimerization with small musculoaponeurotic fibrosarcoma (sMaf) protein and binding to ARE [21]. Besides, Neh1 also promotes the cytoplasmic-to-nuclear translocation of Nrf2 [22]. Neh2 domain is highly conserved between species [23] and enables Nrf2 to combine with Kelch-like ECH-associated protein 1 (Keap1) [24, 25]. Additionally, the presences of lysine residues and a serine residue in Neh2 domain are involved in the suppression of proteasome-mediated Nrf2 degradation [26] and Nrf2-Keap1 complex structure modulation, respectively. The functions of Neh3–Neh6 domains are relatively simple. Briefly, Neh3–Neh5 domains are essential for Nrf2 transcriptional activity [27], and Neh6 domain is associated with Nrf2 degradation [28]. Neh7 domain, which has been identified recently, suppresses Nrf2 downstream gene expression through binding to the retinoic acid receptor *α* [29]. Nrf2 molecular structure is given in Figure 1(a).

Keap1 was first recognized in 1999 with the application of the Neh2 domain of Nrf2 [25]. The Keap1 molecules from different species are slightly different. The number of cysteine residues in the Keap1 molecule from mouse and human beings is 25 and 27, respectively. To be noticed, cysteine residues are sensors for Nrf2 activation [30]. Five domains have been discovered in the Keap1 molecule. They are NTR, BTB, IVR, DGR, and CTR [23]. The BTB domain is required for the homodimerization of Keap1 [31] and the interaction between Keap1 and Cullin3-Rbx1E3 ubiquitin ligase (Cul3-E3-ligase) [32]. The cysteine-rich IVR domain is sensitive to oxidation [33]. Three important cysteine residues (Cys151, Cys273, and Cys288) were identified to be crucial for maintaining the structural integrity of Keap1 [34–36]. The DGR domain is where Keap1 binds to the Neh2 domain of Nrf2 [37]. The Keap1 molecular structure is given in Figure 1(b).

### 3.2. Working Model of Nrf2/ARE Pathway

**Figure 2:**
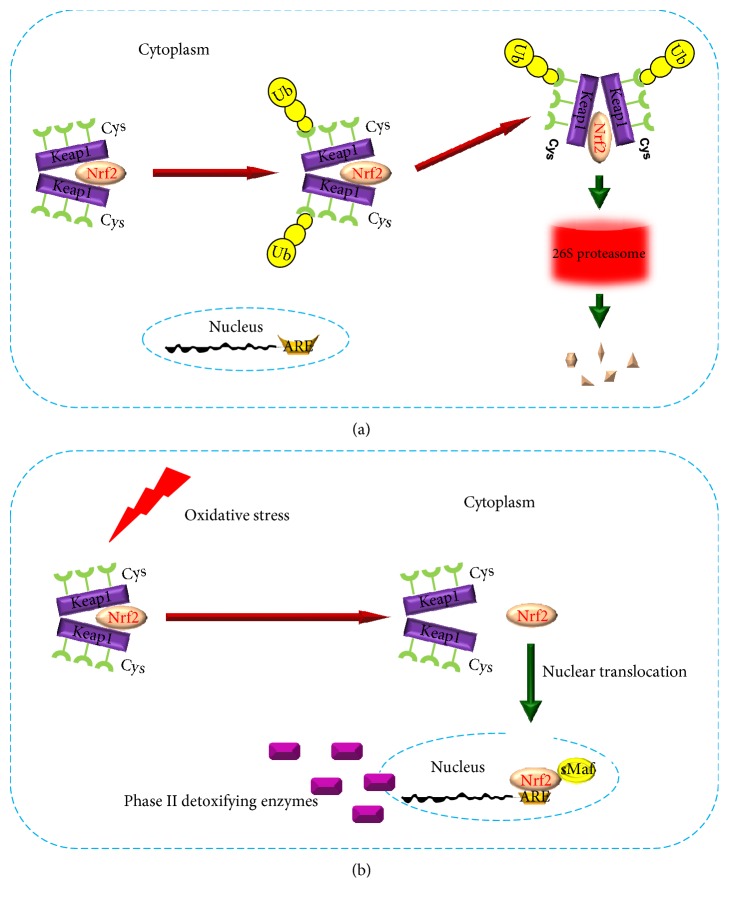
Working model of Nrf2/ARE pathway. (a) Working model of Nrf2/ARE pathway under rest conditions. (b) Working model of Nrf2/ARE pathway during oxidative stress.

Nrf2/ARE pathway plays a crucial role in cellular resistance to oxidative stress [5]. Under rest conditions, Keap1 interacts with Nrf2 to form a complex [38, 39]. At this situation, Keap1 acts as an inhibitor of Nrf2. Moreover, this Nrf2 inhibitor can also interact with Cul3-E3-ligase, which mediates Nrf2 degradation in ubiquitin-proteasome system. After that, the transcription function of Nrf2 is repressed [40] (Figure 2(a)). During oxidative stress, Nrf2 is activated. By modifying the three important cysteine residues of Keap1, conformational changes occur in the Nrf2/Keap1 complex [30, 35], leading to Nrf2 liberalization, followed by Nrf2 translocation. In the nucleus, Nrf2 binds to ARE with the help of sMaf protein [21]. Then, Nrf2 triggers the transcription of phase II detoxification enzymes and antioxidants, such as heme oxygenase-1 and superoxide dismutase 1 [41–43] (Figure 2(b)).

### 3.3. Nrf2/ARE Pathway Regulation

As an executor, Nrf2/ARE signaling pathway is regulated by complex mechanisms [44]. The regulation models include transcriptional level, translational level, and posttranslational level. To examine the mechanisms of Nrf2 transcriptional activation, Kwak et al. found that Nrf2 could autoregulate itself via a specific element of its own promoter [45]. Nowadays, microRNAs are thought to take part in the regulation of oxidative stress [46]. It was suggested that Nrf2 gene could be directly downregulated by miR-28 [47], miR-34a [48], and miR-144 [49]. Additionally, Nrf2 gene could be indirectly regulated by miR-200a-driven Keap1 gene downregulation [50]. Moreover, it is suggested that both DNA methylation [51, 52] and histone modification [53, 54] can regulate Nrf2/ARE pathway. Such information about the epigenetic regulation of Nrf2 and Keap1 was well summarized in a review article [55].

As we talked above, Nrf2 is degraded in ubiquitin-proteasome system. This process keeps Nrf2 protein at a low level and maintains cellular redox homoeostasis by preventing transcription of undesired genes. Therefore, protein level of Nrf2 can be controlled by both regulation of ubiquitin ligases [56] and proteasome activity [57]. In addition to ubiquitin-proteasome system, other proteins can regulate Nrf2 by interacting with the Nrf2-Keap1 complex. For example, autophagy pathway inhibition leads to excessive p62 accumulation. By directly interacting with Keap1, p62 blots Keap1-mediated Nrf2 degradation [58].

Besides regulating transcription and translation, Nrf2 pathway can also be regulated after translation. For example, protein kinase C catalyzes Nrf2 phosphorylation at serine 40 residue, which is critical for promoting Nrf2 separation from Keap1 [59]. Interestingly, p66Shc which takes part in cellular stress response is transcriptionally regulated by Nrf2. However, p66Shc also controls the expression of several Nrf2 downstream genes, like NAD(P)H:quinone oxidoreductase 1 [60]. Taken together, all these signaling pathway regulation methods have the potential to be used for antioxidative stress strategies.

## 4. Role of Nrf2/ARE Pathway in DN: Evidences in Basic Research

The effects of Nrf2/ARE pathway activation on DN in different experimental models were well documented in the past ten years [61]. Sun et al. revealed proper time nodes of Nrf2 signaling pathway in different processes of DN in rats [62]. In addition, hyperglycemia-induced oxidative stress and accelerated renal injury were more serious in streptozotocin-treated Nrf2 knockout mice than those in wild-type controls [63–65], indicating a beneficial effect of Nrf2 on DN. With the expectation that Nrf2-targeted genes would be increased, Keap1 knockout and knockdown mice were generated. However, Keap1 knockout mice died early because of severe defects [66], and Keap1 knockdown mice show different impact effects on metabolic homeostasis [67–69]. The possible reasons for the discrepancy could be the component and content of the diet and the length of high-fat feeding. To be noticed, genetic and pharmacological activation of Nrf2 is constitutive and intermittent, respectively, and the levels of Nrf2 downstream genes in the genetically modified mice were higher than those induced by pharmacological Nrf2 inducers [70]. Moreover, aberrant Nrf2 activation causes unexpected side effects, which will be discussed at the end of this review. Therefore, either pharmacologically activating Nrf2 pathway or inhibiting Nrf2 degradation intermittently might be considered as potential strategies for treating DN.

Recently, a mount of Nrf2 activators have been applied to explore the therapeutic potential of Nrf2 in experimental DN models. In these studies, Nrf2 activators acted upon a series of targets via different mechanisms. The information of these articles was summarized and listed in Table 1 [57, 71–106].

### 4.1. Nrf2-Keap1 Complex Modulation

As a novel Nrf2 activation strategy, directly destroying Nrf2-Keap1 complex has been proved to be effective. For example, under diabetes condition, miR-29 was downregulated. Because miR-29 directly targeted to Keap1 mRNA, Nrf2 content was indirectly reduced [81]. Our previous study demonstrated that C66 (a novel curcumin analogue) ameliorated DN by miR-200a-mediated Nrf2 activation [93]. This method of Nrf2 regulation by miR-200a was confirmed by another study from Wei et al. [107]. Taken together, miR-29 activator and miR-200a activator have the potential to be used in the treatment of DN.

Huang et al. claimed that, in HepG2 cells, PKC promoted Nrf2 phosphorylation at Ser-40. This modulation broke the Nrf2-Keap1 complex and promoted the division between Nrf2 and Keap1 [59]. Therefore, developing medicines inhibiting the interaction between Nrf2 and Keap1-Nrf2 will be a valuable topic.

### 4.2. Reduction of Nrf2 Degradation

The degradation of Nrf2 occurs in the proteasome system. Thus, suppressing the degradation of Nrf2 via inhibiting proteasome activity seems to be a reasonable strategy for DN. Studies from both other and our groups revealed that low dose of MG132, a proteasome inhibitor, had preventive and therapeutic effects on the development and progression of DN in rodents [57, 106].

In addition, minocycline which has been widely used in clinical research is beneficial to the stabilization of endogenous Nrf2 in the kidneys of *db/db* mice, followed by the reduction of glomerular ROS generation. The underlying mechanism might be minocycline could reduce Nrf2 ubiquitination and then decrease its degradation. However, minocycline-mediated amelioration of DN disappeared in diabetic Nrf2 knockout mice [92].

### 4.3. Cytoplasm-to-Nuclear Shuttling of Nrf2

Under certain condition, Nrf2 shuttles from cytoplasm to nucleus. Nucleus accumulation of Nrf2 was proven to be effective against diabetes-induced kidney injury [94]. Using human renal tubular cells, our previous study claimed that Zinc increased the Nrf2 protein level in nucleus and upregulated the expression of Nrf2 downstream enzymes through promoting Akt/GSK-3*β*-mediated inhibition of Fyn, which is a Nrf2 nuclear exporter [98]. Following studies confirmed our viewpoint in type 1 diabetic rodents by using fenofibrate [95] and sulforaphane [96], respectively. Interestingly, it was suggested that sulforaphane mainly induced Nrf2 via modification of Keap1 Cys151 [36]. However, Shang et al. demonstrated that sulforaphane attenuated experimental DN partially through GSK-3*β*/Fyn/Nrf2 pathway [96]. This study provided another possible mechanism underlying the regulation of Nrf2 by sulforaphane. Additionally, Chen et al. found that connexin43 might hinder the nuclear export of Nrf2 by inhibiting c-Src activity and then attenuate renal fibrosis in high glucose-treated glomerular mesangial cells [91]. All these Nrf2 activators promoted Nrf2 nucleus accumulation by blocking Nrf2 export.

Moreover, beneficial effects of facilitating the nuclear import of Nrf2 by other Nrf2 activators, such as hydrogen sulfide [100], hepatocyte growth factor [103], and tert-butylhydroquinone [105], were proved in many experimental DN models, indicating the importance of the regulation of cytoplasm-to-nuclear shuttling of Nrf2 in the treatment of DN.

### 4.4. Other Underlying Mechanisms for Nrf2 Activation

In a recent study, streptozotocin-treated Nrf2 knockout mice and wild-type controls were administrated with or without sodium butyrate for twenty weeks. Data showed that Nrf2 played a key role in the process of sodium butyrate protection against DN. Additionally, sodium butyrate might upregulate Nrf2 at the transcriptional level possibly by inhibiting histone deacetylase activity [90]. As a NAD-dependent histone deacetylase in the nucleus, Sirt1 takes part in many biological processes [108] through working on a battery of transcription factors, including Nrf2 [109–111]. Studies revealed that both resveratrol [101] and its analogue (polydatin) [97] activated Nrf2/ARE pathway through upregulating Sirt1 in glomerular mesangial cells and attenuated high glucose caused fibrosis.

Moreover, Nrf2-dependent beneficial effects on the prevention of DN were observed in low dose of radiation-treated mice [102] and high glucose-treated NRK-52E cells [99], respectively. Activation of Nrf2 in these two studies was reported to be responsible to Akt activation; however, whether Fyn was involved in this process was not mentioned [99, 102].

Oxidative stress aggrieves all kinds of kidney resident cells, including epithelium (podocyte), endothelium, glomerular mesangial cell, and tubular cell. According to Table 1, available evidences support that activation of Nrf2/ARE pathway not only attenuates high glucose-induced podocyte [82, 92, 112, 113], endothelium [71, 76], and mesangial cell [73, 86, 91, 97, 101, 103, 105, 107] injury in glomeruli but also alleviates renal tubular cell [72, 80, 81, 98, 99] damage. In addition, sulforaphane [85, 86, 89, 96] and resveratrol [74, 84, 87, 97, 101] are the most well-studied ones among those Nrf2 activators. Besides, curcumin and its analogues (C66) were also introduced in three studies [78, 80, 93] and have emerged to be a potential therapeutic choice, which will be discussed later in this review.

Taken together, breaking the Nrf2-Keap1 complex, blocking Nrf2 degradation, and promoting Nrf2 cytoplasmic-to-nuclear translocation are valuable strategies to increase antioxidant generation. These basic evidences provide us with new ideas for the treatment of oxidative stress-associated kidney injury under diabetes condition.

## 5. Role of Nrf2/ARE Pathway in DN: Evidences in Clinical Research

Beneficial effects of Nrf2/ARE pathway activation on DN can be observed in not only basic researches but also clinical researches. Additionally, the relationship of genetic variants of Nrf2 (rs2364723, rs10497511, rs1962142, and rs6726395) and diabetes complications in Han descents of Northeast China has been confirmed recently by our group [114]. Moreover, dysregulation of Nrf2 signaling was also observed in human diabetes kidney [64]. Thus, Nrf2 activators began to be used in patients with DN in clinical trials.

The most well-studied promising candidate Nrf2 activator is bardoxolone methyl (BM), which has been used not only for cancer [115] but also for many other oxidative stress and inflammation-involved chronic diseases [116]. BM was first introduced to clinicians because it possesses anticancer effects. When treating patients who suffered from malignant diseases, the investigators surprisingly found that BM could raise estimated GRF (eGFR), accompanied by increasing NQO1 mRNA in peripheral blood mononuclear cells. Interestingly, these improvements were more likely to be observed in a subset of patients with CKD [117, 118]. Based on these observations, this multifunctional medicine was thought to ameliorate renal damage in type 2 diabetes patients.

With the encouraging idea, BM was further studied in a phase 2a clinical trial to evaluate its renoprotective efficacy and safety [119]. In this multicenter clinical trial, short-term (8 weeks) effects of BM on kidney function was evaluated in type 2 diabetes patients and moderate to severe CKD (baseline serum creatinine level ranged from 1.3 to 3.0 mg/dl). All enrolled patients were administrated with 25 mg BM for 4 weeks and 75 mg BM for next 4 weeks. Data showed that the administration of BM obviously increased eGFR and creatinine clearance while decreased serum creatinine and blood urea nitrogen. These results seemed very optimistic; however, two major limitations in this study should not be ignored. First, this was a self-control study and a placebo control group was needed to obtain a more convincing result. Second, the treatment course was not long enough to observe side effects.

Attempting to further verify the beneficial effects of BM in patients who suffered from diabetes and CKD, the BEAM study with a larger sample size was conducted [120]. To overcome the limitations in the previous phase 2a clinical trial, 227 patients who suffered from diabetes mellitus and CKD were enrolled in the BEAM study. Participants were administrated with either placebo or different doses (25, 75 or 150 mg/day) of BM for 52 weeks. Data showed that BM ameliorated eGFR, which persisted during the 52-week treatment period. Moreover, adverse events reported in the BEAM study were moderate and manageable.

After obtaining these exciting results, the BEACON study was performed in June 2011 [121]. The purpose of this study was further confirming the beneficial effects of BM on safely reducing renal and cardiac morbidity and mortality among patients who suffered from diabetes mellitus and CKD. More than two thousands patients who suffered from type 2 diabetes mellitus and stage 4 CKD were recruited and randomly administrated with either placebo or BM (20 mg/day) for 52 weeks. However, the BEACON study was stopped prematurely in October 2012 due to safety concerns about considerable adverse events as well as high mortality rates. Heart failure events (hospitalization or death) occurred in 96 from 1088 patients and 55 from 1097 patients in the BM group and placebo group, respectively. Moreover, composite outcome events (including nonfatal stroke and myocardial infarction, hospitalization because of heart failure, and death because of cardiovascular diseases) occurred in 139 from 1088 patients and 86 from 1097 patients in the BM group and placebo group, respectively. Compared to the placebo group, although eGFR was improved, urinary protein level and blood pressure were also obviously increased in the BM group during the study period.

The main reason for the termination of the BEACON study was high rates of heart failure, and many occurred early after BM initiation. Analyses aiming to find the reasons for heart failure events in the BEACON study were conducted [122, 123]. Chin et al. claimed that both high B-type natriuretic peptide level and heart failure-caused hospitalization before were risk factors of heart failure [122]. Another mechanism underlying cardiovascular events in the BEACON study was also revealed by Chin et al. It was suggested that BM might pharmacologically lead to acute water-sodium retention and hypertension by modulating the endothelin pathway [123]. Besides heart failure events, hypertension was also more likely to happen in patients from the BM group than from the placebo group. There were two possible reasons to explain this phenomenon. First, endothelin pathway regulation by BM leads to sodium and volume retention as we described above. Second, by binding to the promoter of intrarenal angiotensinogen gene, Nrf2 stimulates its expression and then activates the renin-angiotensin system [124]. Therefore, BM might lead to hypertension by both sodium and volume retention and renin-angiotensin system activation.

In the BEACON study, investigators did observed elevated eGFR in the BM group. This phenomenon was possibly due to a similar structure between BM and cyclopentenone prostaglandins, which can cause renal vasodilatation. To be noticed, eGFR was calculated by serum creatinine in the BEAM study and muscle mass will influence the level of serum creatinine [125, 126]. Compared to those in the placebo group, the degree of weight loss and muscle wasting in the BM group was higher, which might cause an overestimation of eGFR in the BM group [127].

Although some adverse events occurred in BM-treated patients, it cannot stop us from exploring new medicines for the treatment of DN. Different to BM, curcumin was proven to be effective in a DN experiment model [78, 80, 93]. In a recent clinical trial with small sample size, 14 patients with diabetic kidney disease were given curcumin (500 mg/day) for 2–4 weeks. It was claimed that short-term curcumin intervention attenuated kidney damage partially by activating Nrf2 pathway [128], which enhanced our confidence.

## 6. Other Mechanisms of Nrf2 in the Treatment of DN

### 6.1. Effects of Nrf2 Activation on Anti-Inflammation

Besides having an antioxidant property, Nrf2 was proven to alleviate inflammation, which is also involved in the progression of DN. Alleviation of kidney damages accompanied by downregulation of inflammatory cytokines (monocyte chemotactic protein-1, intercellular cell adhesion molecule-1) was observed in digitoflavone-treated wild-type diabetic mice instead of Nrf2 knockout diabetic mice [79]. However, the mechanism under which Nrf2 regulated these two proinflammatory mediators was not mentioned. Another study revealed direct evidence of inflammation suppression by Nrf2. Kobayashi et al. demonstrated that Nrf2 interfered with transcriptional upregulation of IL-6 and IL-1b by binding to these two genes and inhibiting RNA polymerase II recruitment [129].

### 6.2. Effects of Nrf2 Activation on Antifibrosis

Fibrosis is an ultimate pathway in the pathogenesis of DN, and TGF-*β*1 advances kidney fibrosis. It was suggested that Nrf2 ameliorated DN by transcriptional repression of TGF-*β*1 directly. Gao et al. found that Nrf2 inhibited TGF-*β*1 through binding to the specific region in TGF-*β*1 promoter with the help of c-Jun as well as SP1 [130]. This study provided a novel clue for DN prevention and intervention.

## 7. Side Effects of Nrf2 Activation

Considerable evidence showed beneficial effects of Nrf2 on DN. However, side effects of this molecule should also be noticed. Zoja et al. treated type 2 diabetic rats with a synthetic triterpenoid analogue of BM (RTA405). They demonstrated that RTA405 elevated mortality, aggravated proteinuria, dyslipidemia, kidney function, and liver function [131]. These findings indicated the toxicity of this molecule. In addition, by using another BM analogue (dh404), the deleterious and salutary actions of Nrf2 activation in the treatment of DN were reported to be dose-dependent. Low dose of dh404 might alleviate kidney damage, while high dose of dh404 might lead to deterioration of the kidney function [132, 133]. Therefore, consideration of the appropriate dose is necessary when applying the BM analogues.

Dural roles of Nrf2 in cancer have also been reported [134, 135]. Some studies suggested that aberrant Nrf2 activation was correlated with cancer progression. For example, one important mechanism of hereditary leiomyomatosis and renal cell carcinoma is fumarate hydratase inactivation-induced fumarate accumulation. Sustained fumarate accumulation leads to aberrant Nrf2 activation and then gives rise to fatal consequences [136]. Nrf2 contributes to cancer cell survival not only by upregulating the expression of Bcl-xl and Bcl-2 to inhibit apoptosis [137] but also by raising the rate of glycolysis to promote cell proliferation [138].

Intrarenal renin-angiotensin system causes blood pressure elevation and kidney damage. Another side effect of Nrf2 activation that should be noticed is that Nrf2 can activate the renin-angiotensin system directly [139]. For example, Abdo et al. found that overexpression of Nrf2 promoted angiotensinogen gene transcription in high glucose-treated renal proximal tubule cells. Moreover, application of Nrf2 siRNA reduced angiotensinogen mRNA and protein expression [124].

## 8. Conclusions

In summary, oxidative stress is an important pathogenesis of DN. As the center of the body antioxidant system, Nrf2/ARE pathway is of great value in the treatment of DN. Modulating Nrf2/ARE pathway through different mechanisms provides new approaches for future clinical research studying the treatment of DN. It was worth noting that there was no evidence to support the application of BM in animal DN before the BEACON study. Therefore, before designing a new clinical trial, investigators should make sure that preliminary studies have been done and should not ignore any negative outcome. Moreover, in order to avoid the occurrence of a large number of serious adverse events, possible side effects of Nrf2 activation need to be monitored during the period of trial conduction.

## Figures and Tables

**Table 1:** Beneficial effect of Nrf2 activators on experimental DN.

Nrf2 activator	DN model	Results	Ref.
Sulforaphane	T1DM mice; human renal tubular cells	Sulforaphane alleviates renal inflammation, oxidative stress, fibrosis, and dysfunction in DN mice via activation of Nrf2/ARE pathway (HO-1, SOD1, etc.); beneficial effects disappeared when Nrf2 siRNA was applied	[85]
Sulforaphane	T1DM mice; human renal mesangial cells	Sulforaphane normalizes diabetes-induced kidney oxidative damage, fibrosis, and apoptosis, which is mediated by Nrf2/ARE pathway (NQO1, rGCS, and MRP2) activation; beneficial effects disappeared in Nrf2 knockout mice	[86]
Sulforaphane	T2DM mice	Sulforaphane improves kidney oxidative damage, inflammation, and fibrosis in diabetic mice, accompanied by increasing kidney Nrf2 and its downstream gene metallothionein; beneficial effects disappeared in Nrf2 knockout mice	[89]
Sulforaphane	T1DM rats	Sulforaphane ameliorates DN through GSK3*β*/Fyn/Nrf2 signaling pathway (prevents nuclear export of Nrf2)	[96]
Resveratrol and rosuvastatin	T1DM mice	Resveratrol combined with rosuvastatin treatment normalizes the TGF-*β*1, FN, and NF-*κ*B/p65 and restores Nrf2 in renal tissues of diabetic rats	[74]
Resveratrol	T1DM rats; rat mesangial cells	Resveratrol reduces albuminuria and mesangial matrix expansion in DN rats and attenuates mesangial cell proliferation, which is associated with upregulation of Nrf2 and glutathione S-transferases Mu	[84]
Resveratrol	T1DM rats	Resveratrol protects against DN by alleviating oxidative damage and inflammation through Nrf2/ARE pathway (SOD, CAT, etc.)	[87]
Resveratrol	Rat primary glomerular mesangial cells	Resveratrol inhibits AGE-induced FN and TGF-*β*1 in glomerular mesangial cells through Sirt1/Nrf2 signaling pathway activation	[101]
Polydatin (resveratrol analogue)	T1DM rats; rat glomerular mesangial cells	Polydatin inhibits AGE-induced FN and TGF-*β*1 in glomerular mesangial cells is associated with activation of Sirt1/Nrf2 pathway	[97]
Curcumin	T2DM rats	Curcumin ameliorates albuminuria, kidney pathophysiologic changes, and urinary MDA, accompanied by increasing Nrf2, HO-1, and urinary SOD	[78]
Curcumin	Rat kidney tubular epithelial cells	Curcumin protects renal tubular cells from high glucose-induced EMT through upregulating Nrf2 and HO-1; beneficial effects disappeared when Nrf2 siRNA was applied	[80]
C66 (curcumin analogue)	T1DM mice	C66 protects against DN by upregulating Nrf2 via both increasing miR-200a and inhibiting miR-21; beneficial effects were partially abolished in Nrf2 knockout mice	[93]
Zinc	Human renal tubular cells	Zn sensitizes Nrf2 by facilitating Akt-associated Fyn inhibition (prevents Nrf2 nuclear export) and thus alleviates kidney oxidative and inflammatory damage and fibrosis	[98]
Zinc	Rat kidney tubular epithelial cells	Zinc ameliorates high glucose-mediated apoptosis in rat kidney tubular cells through Akt/ERK/Nrf2 signaling pathway activation (promotes Nrf2 accumulation in nuclear)	[99]
MG132	T1DM mice; human renal tubular cells	MG132 sensitizes Nrf2 by inhibiting proteasome activity and thus attenuates hyperglycemia-induced kidney oxidative and inflammatory damage, fibrosis, and eventual dysfunction; beneficial effects disappeared when Nrf2 siRNA was applied	[57]
MG132	T1DM rats	Low dose of MG132 prevents diabetes-induced kidney damage by Nrf2/ARE pathway activation	[106]
Rutin	Human renal glomerular endothelial cells	Rutin significantly prevents hyperglycemia-induced glomerular endothelial barrier disruption by decreasing ROS through the activation of Nrf2	[71]
Berberine	T1DM mice; rat renal tubular epithelial cells	Berberine ameliorates high glucose-induced EMT and oxidative stress by Nrf2/ARE pathway (HO-1 and NQO1) activation and TGF-*β*/EMT pathway inhibition; beneficial effects disappeared when Nrf2 siRNA was applied	[72]
Casein kinase 2 interacting protein-1	Rat glomerular mesangial cells	Casein kinase 2 interacting protein-1 downregulates ICAM-1 and FN by Nrf2/ARE pathway (SOD1 and HO-1) activation	[73]
Salvianolic acid A	T1DM mice	Salvianolic acid A protects DN via Nrf2/ARE pathway (HO-1, NQO1, and GPx-1)	[75]
Sinomenine	Human renal glomerular endothelial cells	Sinomenine reduces ROS level and exerts renal protective effect by activating Nrf2 in high glucose-treated human renal glomerular endothelial cells	[76]
*Momordica charantia* polysaccharides	T1DM rats	*Momordica charantia* polysaccharides attenuate type 1 DN in rats by upregulating Nrf2, CAT, GSH, and SOD	[77]
Digitoflavone	T1DM mice	Digitoflavone minimizes pathological changes, decreases oxidative and inflammatory damage as well as fibrosis in DN mice, which is mediated by Nrf2 pathway (GCLC and HO-1) activation; beneficial effects disappeared in Nrf2 knockout mice	[78]
Thrombomodulin domain 1	T2DM mice	Thrombomodulin domain 1 improves DN by suppressing inflammation, activating the Nrf2 pathway, and inhibiting apoptosis in the mouse kidney	[82]
Maxacalcitol	T2DM mice	Maxacalcitol alleviates DN by suppressing kidney oxidative and inflammatory damage as well as fibrosis, which is mediated by Nrf2 pathway (GCLC and HO-1) activation	[83]
4-Phenylbutyric acid	T1DM rats	Treatment with 4-phenylbutyric acid attenuates oxidative damage in DN rats via Nrf2 facilitation	[88]
Sodium butyrate	T1DM mice	Sodium butyrate protects against DN through Nrf2 upregulation, which is mediated by suppressing HDAC function; beneficial effects disappeared in Nrf2 knockout mice	[90]
Connexin43	Primary glomerular mesangial cells; type 2 diabetic mice	Connexin43 activates Nrf2/ARE pathway by means of inhibiting c-Src activity to hinder the nuclear export of Nrf2 and then downregulates FN, ICAM-1, and TGF-*β*1 expression and ultimately attenuates renal fibrosis in diabetic mice	[91]
Minocycline	T1DM/T2DM mice; human/mouse podocytes	Minocycline stabilizes endogenous Nrf2 by reducing its ubiquitination and reduces markers of oxidative damage, thus alleviated DN; beneficial effects disappeared in Nrf2 knockout mice	[92]
Mycophenolate mofetil	T1DM rats	Mycophenolate mofetil attenuates DN at least in part by upregulating Nrf2 pathway (increases the nuclear accumulation of Nrf2)	[94]
Fenofibrate	T1DM mice	Fenofibrate attenuates DN via increasing FGF21 and activating Akt/GSK-3*β*/Fyn/Nrf2 pathway (prevents Nrf2 nuclear export)	[95]
Hydrogen sulfide	T1DM rats; rat glomerular mesangial cells	Hydrogen sulfide alleviates DN by suppressing oxidative stress (promotes Nrf2 accumulation in nuclear), inflammation, and renin-angiotensin system activity, as well as by reducing mesangial cell proliferation	[100]
Low-dose radiation	T1DM mice	Prevention of low-dose radiation against DN is associated with Akt phosphorylation and Nrf2 upregulation	[102]
Hepatocyte growth factor	Rat mesangial cells	Hepatocyte growth factor ameliorates high glucose-induced oxidative damage in rat mesangial cells by upregulating 8-nitro-cGMP production, accompanied by nuclear accumulation of Nrf2	[103]
Telmisartan	T2DM mice	Telmisartan inhibits NAD(P)H oxidase and upregulates Nrf2 and SOD, leading to the attenuation of diabetes-induced renal damage	[104]
tert-butylhydroquinone	T1DM mice	tert-butylhydroquinone reduces renal damage through nuclear accumulation of Nrf2 as well as its target genes in type 1 diabetic mice	[105]
